# Incidence of thyroid nodules in early stage autosomal polycystic kidney disease

**DOI:** 10.1186/s12882-022-02714-w

**Published:** 2022-03-03

**Authors:** Ewa Zalewska, Sonia Kaniuka-Jakubowska, Piotr Wiśniewski, Magdalena Jankowska, Krzysztof Sworczak, Alicja Dębska-Ślizień

**Affiliations:** 1 Department of Endocrinology and Internal Medicine, Medical University of Gdansk, Gdansk, Poland; 2 Department of Nephrology, Transplantology and Internal Diseases, Faculty of Medicine, Medical University of Gdansk, Gdansk, Poland

**Keywords:** ADPKD, Polycystic kidney disease, Thyroid nodules, Ultrasonography

## Abstract

**Background:**

Autosomal dominant polycystic kidney disease (ADPKD) is the most common hereditary kidney disease. Defect in cilia-mediated signaling activity is a crucial factor leading to cyst formation. Hence, ADPKD is regarded as a systemic disorder with multiple extrarenal complications, including cysts in other organs, for instance, the liver, pancreas, spleen, or ovaries. Interestingly, loss-of-function of primary cilia has been recently found to contribute to a malignant transformation from degenerated thyroid follicles. However, the increased incidence of thyroid nodules in ADPKD patients has not yet been fully confirmed.

**Objectives:**

To determine the incidence of thyroid lesions in patients with ADPKD in comparison to previous population studies. Moreover, we aimed to investigate if the pace of the disease progression is associated with a higher prevalence of thyroid lesions.

**Material and methods:**

In 49 early-stage ADPKD patients recruited from our center, we performed ultrasonography of the thyroid glands, and laboratory evaluation of thyroids function. We compared the results with population studies.

**Results:**

Twenty-three individuals had solid, cystic-solid, or cystic lesions revealed in the ultrasonography and 2 patients had a positive past medical history for thyroidectomy due to nodular goiter. In 10 patients out of the 23, only minor cysts with no clinical significance were found and 13 out of the 23 patients had solid or cystic-solid lesions, which occurred to be benign based on three years of follow-up or the biopsy of the nodule.

**Conclusions:**

We found no increased incidence of thyroid gland lesions in early ADPKD patients in comparison to previous population studies. Plausibly, mechanisms other than defective cilia signaling are involved in the risk for focal thyroid lesions formation. Moreover, the rate of progression of kidney function decline seems to be not accompanied by the higher incidence of thyroid pathology.

## Introduction

Autosomal dominant polycystic kidney disease (ADPKD) is the most common hereditary kidney disease [1]. ADPKD results primarily from a mutation in one of two genes, *PKD1* or *PKD2*, which encoded proteins, respectively, polycystin-1 (PC1) and polycystin-2 (PC2). These proteins are localized in the cilium, a solitary structure present on nearly every cell in the mammalian body. A defect in cilia-mediated signaling activity, caused by the deficiency of PC1 and PC2, is a crucial factor leading to cyst formation [2]. Hence, ADPKD is regarded as a systemic disorder with multiple extrarenal complications, including cysts in other organs, for instance, the liver, pancreas, spleen, or ovaries [1].

The most prevalent life threatening complication of ADPKD is end-stage kidney disease (ESKD). Interestingly, the age at which patients with ADPKD will reach ESKD shows huge interindividual variability [3]. It is not clear weather rapid kidney function decline is accompanied by more pronounced external manifestations of the disease. Kidney length is regarded as sufficient to stratify the risk of progression to renal insufficiency early in ADPKD. The ultrasound kidney length over 16.5 cm has the best cut-point for predicting the development of chronic kidney failure (CKD) stage 3 [4]. Moreover, patients may be classified as having either rapidly or slowly progressive disease based on the average annual change in estimated glomerular filtration rate (eGFR) [3].

Unquestionably, there are several interactions between thyroid and kidney functions. It has been reported that CKD patients have an increased incidence of non-thyroidal illness (so-called ‘low T3 syndrome’) and subclinical or primary hypothyroidism [5–8]. The potential mechanisms of reduced peripheral conversion of thyroxine (fT4) to triiodothyronine (fT3) including inhibition of expression of type 1 5′-deiodinase by inflammatory cytokines, affected protein binding of fT3 due to chronic protein malnutrition, and inhibition of thyroid iodide organification, so-called Wolff-Chaikoff effect, caused by impaired renal handling of iodine.

Interestingly, loss-of-function of primary cilia has been recently found to contribute to a malignant transformation from degenerated thyroid follicles [9]. However, the increased incidence of thyroid nodules in ADPKD patients has not yet been fully confirmed [8, 10–12]. In a small study of 30 ADPKD patients, incidence of thyroid cysts was reported to be at 6.7% [10] and case of multiple thyroid cysts has been reported in one ADPKD patient [11]. So far, the incidence of various thyroid nodules, including malignant ones, in ADPKD patients has not been studied.

Thyroid nodules may be detected in ultrasonography in 19%–68% of randomly selected individuals and thyroid cancer occurs in 7%–15% of cases depending on age, sex, radiation exposure history, and family history [13]. The vast majority of thyroid cancers are solid or mostly solid with minimal (<5%) cystic component [13]. The pattern of sonographic features combined with the nodule size determine the necessity of biopsy of an incidentally detected thyroid nodule [13].

The aim of the study was to determine the incidence of thyroid lesions in patients with ADPKD in comparison to previous population studies. Moreover, we aimed to investigate if the pace of the disease progression (rapid vs. non-rapid) is associated with a higher prevalence of thyroid lesions.

## Methods

### Study subjects

To analyze the thyroid morphology and function in ADPKD, we conducted a prospective study including 49 ADPKD patients, who attended our outpatient clinic of Nephrology in 2017-2020. Of the two hundred and twenty-two consecutive patients who were over eighteen years of age and fulfilled ADPKD Ravine-Pei diagnostic criteria, one hundred and seventy-three patients met exclusion criteria, which were stage 3b to 5 of CKD and the patient's lack of consent.

We classified patients with normal kidney function based on eGFR > 60 ml/min/1.73m^2^ as slow or rapid progressors according to ultrasound kidney length below or over 16.5 cm, respectively. Moreover, we grouped the patients with or without polycystic liver disease.

### Study design

From each patient, medical history was obtained, including symptoms and signs consistent with hyper- and hypothyroidism, family history, and current medication usage. Afterward, everyone underwent neck palpation, thyroid ultrasonography, and laboratory evaluation of the function of thyroid gland (thyroid-stimulating hormone (TSH), fT4, fT3, anti-thyroid peroxidase antibodies (TPOAb), and thyroglobulin antibodies (TgAb)) and kidney (serum creatinine concentration and eGFR). Moreover, the patients diagnosed with thyroid nodules underwent a biopsy of the lesion or at least a 3-years follow-up in the outpatient Endocrinology Clinic.

We compared subjects from our cohort with solid and cystic-solid thyroid lesions and without positive past medical history for thyroid diseases or symptoms of hypo- or hyperthyroidism to a population study from the same region of Gdansk, Sopot, and Gdynia conducted by Karaszewski et al. [14]. Karaszewski et al. performed in 2006 neck palpation, thyroid gland ultrasonography, and TSH level measurement in one hundred thirty-five healthy adults (ninety-five women and forty men) [14]. The cohort in Karaszewski et al. study and our cohort were in comparable age, male to female ratio, and from the same region (low iodine deficiency and similar exposure to ionizing radiation). In the study by Karaszewski et al. minor (< 1 cm), pure cysts in thyroid glands have not been reported. Therefore, we also did not take into account those minor, pure thyroid cysts in our patients when comparing the number of thyroid nodules in both cohorts.

### Ultrasonography of the thyroid

An endocrinologist with over ten years of experience in thyroid sonography examined all patients, with a linear probe of 10 MHz frequency, using GE Logiq 5 Ultrasound System.

### Volume of thyroid

We evaluated the thyroid gland volume based on the use of an ellipsoid model with Brunn's modification [15]. The height, width, and depth of each lobe (always selecting the largest of the obtained dimensions) were measured and multiplied. The result was then multiplied by an empirically determined correction factor of 0.479.

$$Thyroid\;lobe\;volume=height\;x\;width\;x\;depth\;x\;0.479$$

Subsequently, we added together volumes of both thyroid lobes, not including the volumes of the isthmus in the calculations. The calculation method was consistent with that used in Karaszewski's et al. study.

### Volume of thyroid nodules

The volumes of the thyroid nodules were calculated based on the use of an ellipsoid model. The height, width, and depth, always selecting the largest of the obtained dimensions, were measured and multiplied. Finally, the results were multiplied by a correction factor π/6 (~0.524).

$$Thyroid\;nodule\;volume= height\;x\;width\;x\;depth\;x\;\frac{\uppi}{6}$$

### Sonographic follow-up and fine needle aspiration

Thyroid nodules ≥1 cm in greatest dimension with suspicion sonographic pattern for malignancy were classified for fine needle aspiration [13]. Patients after fine-needle aspiration and those with nodules that did not meet criteria for fine-needle aspiration at initial imaging were sonographic followed-up in accordance with the American Thyroid Association Management Guidelines for Adult Patients with Thyroid Nodules [13].

### Laboratory measurements

TSH, fT4, fT3, TPOAb, and TgAb concentrations in serum were determined in Central Clinical Laboratory in Gdansk using the chemiluminescent microparticle immunoassay (CMIA) method (Alinity I Abbott Laboratories Poland). Moreover, serum creatinine concentration has been measured by the spectrophotometric method (Alinity C Abbott Laboratories Poland). The Chronic Kidney Disease Epidemiology Collaboration (CKD-EPI) eGFR has been calculated based on the creatinine concentration.

### Statistic

Data were prepared using Excel software and analyzed using R-studio. For continuous quantitative data ranges, mean, median, standard deviation (SD), percentiles (Q – Q3), and density plots were depicted. Chi-squared test or Fisher test was applied for the analysis of qualitative variables depending on the sample size. Welch's t-test or Mann–Whitney U test were used for quantitative variables depending on the data distribution. We used the Shapiro–Wilk test to determine if a data set is well-modeled by a normal distribution. All analyses were adjusted for confounding factors like age and sex. A *p*-value of <0.05 was considered significant.

## Results

Forty-nine ADPKD patients aged 19 to 64 were included, among whom 40 were women (81.6%) and 9 were men (18.3%). The clinical characteristics of the subjects are shown in Table 1.

**Table 1:** The clinical characteristics, laboratory values, and thyroid ultrasound results of our cohort

Patient number	49
Male/female	9/40
Age [years] – mean (SD)	36.7 ± 11.9
Number of patients with solid thyroid nodules	6
Number of patients with cystic-solid thyroid nodules	7
Number of patients with cystic lesions	10
Number of patients after thyroidectomy due to nodular goiter	2
Number of patients with Hashimoto disease	1
Number of patients with thyrotoxicosis	1
Serum creatinine concentration [mg/dl] - median (Q – Q3)	0.82 (0.68 – 0.96)
eGFR [ml/min/1.73 m^2^] - mean (SD)	100 ± 22
TSH [mlU/l] - mean (SD)	1.2 ± 0.64
Ft3 [pmol/l] - median (Q – Q3)	4.2 (3.78 – 4.66)
Ft4 [pmol/l] - median (Q – Q3)	12.6 (11.6 – 13.8)
Number of TgAb-positive patients	21
Number of TPOAb -positive patients	8

*eGFR* estimated glomerular filtration rate, *fT3* triiodothyronine, *fT4* thyroxine, *(Q1 – Q3)* quartile range, *SD* standard deviation, *TgAb* thyroglobulin antibodies, *TPOAb* anti-thyroid peroxidase antibodies, *TSH* thyroid-stimulating hormone

Based on medical history, two patients from our cohort underwent thyroidectomy due to the nodular goiter. Hashimoto disease was present in one individual, and one patient had symptoms of hyperthyroidism (Table 1). Thirteen subjects had a positive family history of thyroid disease, and forty-one patients had a positive family history of ADPKD. The neck palpation revealed a thyroid nodule in only one patient. Mean serum TSH concentration was 1.2 μU/ml (Table 1). FT3 and fT4 were within referral ranges except for one patient who had elevated fT3 and, based on the clinical symptoms and additional examination, was diagnosed with thyrotoxicosis. Twenty-one patients had elevated TgAb (42.86%), and eight patients had positive TPOAb (16.33%) (Table 1). All our patients had eGFR above 60 ml/min/1.73 m^2^.

The ultrasonography revealed thyroid nodules in twenty-three patients. In ten patients, only minor cyst without clinical significance (less than 5 mm) were found and thirteen patients had solid or cystic-solid lesions. Only one patient required fine needle aspiration of the thyroid nodule and the lesion occurred to be benign (category II in The Bethesda System for Reporting Thyroid Cytopathology [16]). Thyroid nodule of the patient after fine needle aspiration and patients with thyroid lesions, which did not meet criteria for fine needle aspiration, were sonographic followed-up in line with the American Thyroid Association Management Guidelines for Adult Patients with Thyroid Nodules [13]. The thyroid nodules did not change during the 3-years of follow-up.

The mean volume of the thyroid gland in our ADPKD female patients and men patients was 10.1 mL and 16.9 mL, respectively (the referral ranges for thyroid volume are 10 to 15 mL for adult females and 12 to 18 mL for adult males [17]). The presence of thyroid nodules did not differ significantly with sex (Table 2).

**Table 2:** Comparison of thyroid ultrasound results and clinical characteristics of ADPKD patients in our cohort according to gender

	**Female** (*n* = 40)	**Male** (*n* = 9)	***p*-value**
Age [years] – mean (SD)	35.6 ± 10.6	41.6 ±16.7	0.333
V of TG [ml] - mean (SD)	10.1 ± 2.64	16.9 ± 8.43	0.057
n with TN (%**)**	18 (45%)	6 (67%)	0.289
Mean V of TN [ml] - median (Q – Q3)	0.0122 (0.005-0.145)	0.08 (0.052-0.156)	0.386

*P*-values were calculated using independent-sample t-test in the case of normal distribution and Mann-Whitney U in case of non-normal distribution. For categorical data, the Fisher test was used due to the small sample size. *n* number of patients, *SD* standard deviation, *V* volume, *TG* thyroid gland, *TN* thyroid nodules, *(Q1 – Q3)* quartile range

We classified twenty patients from our cohort as fast progressors and twenty-nine patients as slow progressors based on the ultrasound kidney length over or below 16.5 cm, respectively. Thirty-one out of the forty-nine patients had polycystic liver disease (Table 3).

**Table 3:** Comparison results of the number of thyroid nodules and mean volume of thyroid nodules of patients with polycystic liver disease or not and according to the kidney size above 16,5 cm (rapid progressors) or below 16,5 cm (slow progressors)

	**Liver cysts **(*n* = 31)	**No liver cysts **(*n* = 18)	***p*-value**
n with TN (%)	15 (48%)	9 (50%)	1
Mean V of TN [ml] - median (Q – Q3)	0.047 (0.006 – 0.15)	0.024 (0.009 - 0.143)	0.765
	**Kidney size > 16.5 cm** (*n* = 20)	**Kidney size < 16.5 cm** (*n* = 29)	***p*-value**
n with TN (%)	10 (50%)	14 (48%)	1
Mean V of TN [ml] - median (Q – Q3)	0.014 (0.007 – 0.085)	0.052 (0.009 – 0.155)	0.478

*P*-values were calculated using independent-sample t-test in the case of normal distribution and Mann-Whitney U in case of non-normal distribution. For categorical data, the chi-square test with Yates’s correction was used. *n* number of patients, *SD* standard deviation, *V* volume, *TG* thyroid gland, *TN* thyroid nodules, *(Q1 – Q3)* quartile range

Renal enlargement and the presence of liver cysts, did not appear to affect the size of the thyroid gland lesions (Table 3).

We compared the results of thyroid volume, the mean thyroid nodules volumes, the number of thyroid nodules, and TSH level in forty-five subjects from our cohort, who had no past medical history for thyroid diseases or symptoms of hypo- or hyperthyroidism with a population study from the region [14] in Table 4.

**Table 4:** The comparison of our patients and cohort from the Karaszewski et al. population study [14]. For the comparison, we excluded from our cohort four patients with a history of thyroid disease

	**Our cohort** **(*n* = 45)**		**Population study** **(*n* = 135)**		***p*-value**	
Sex ratio (female/male)	37/8		95/40		0.173^a^	
Age [years] – mean (SD)	f1 35.5 ± 10.1	m1 40.5 ±17.5	f2 37.0 ± 12.5	m2 41.3 ± 13.55	f1 vs. f2 0.384	m1 vs. m2 0.9
V of TG [ml] – mean (SD)	f1 10.2 ± 2.67	m1 16.9 ± 8.43	f2 15.67 ± 7.26	m2 22.31 ± 13.03	f1 vs. f2 0.000	m1 vs. m2 0.124
TSH [mlU/l] – mean (SD)	f1 1.27 ± 0.648	m1 0.94 ± 0.557	f2 1.87 ± 1.39	m2 1.55 ± 0.83	f1 vs. f2 0.000	m1 vs. m2 0.054
n w/ TN (%)	11 (22%)		32 (23.7 %)		1	
n w/ MTN (%)	6 (12%)		16 (12 %)		1	
Mean V of TN [ml] - median (Q – Q3)	0.141 (0.08 – 0.156)		0.67		0.0039	

*P*-values for our cohort versus cohort from population study are calculated using independent-sample t-test in case of normal distribution, Mann-Whitney U in case of non-normal distribution, and chi-square test with Yates’s correction in case of categorical data. *n* number of patients, *SD* standard deviation, *TG* thyroid gland, *f1* female patients from our cohort, *f2* female subjects form Karaszewski et al. study, *m1* male patients from our cohort, *m2* male subjects form Karaszewski et al. study, *f1 vs. f2* the comparison of the groups of women, *m1 vs. m2* the comparison of the groups of men, *V* volume, *TN* thyroid nodules, *MTN* multiple thyroid nodules, *TSH* thyroid-stimulating hormone. ^a^the comparison of the sex ratio in both cohorts

The percentage of people with thyroid nodules generally (*p* = 1) and multiple thyroid lesions (*p* = 1) did not differ significantly between our cohort and the cohort from the population study [14]. The mean volume of all kind of thyroid nodules (solid, cystic-solid, and cystic) in our cohort was 0.096 mm^3^ and ranged from 0.00124 mm^3^ to 2.88 mm^3^. Unexpectedly, both the volume of the thyroid gland and the volume of thyroid nodules were smaller in our patients than in the population study patients (*p* < 0.05) (Table 4). However, diameters of thyroid nodules were comparable (between 1mm to 25mm in our cohort and between < 3mm to 23mm in the population study).

## Discussion

We found no increased incidence of thyroid lesions as well as thyroid neoplasia in ADPKD patients in comparison to previous population studies [14].

The prevalence of thyroid nodules determined by ultrasonography ranges from 5.2% to 67.0%, depending on the studied population [14, 18, 19]. Most studies indicate that thyroid nodules are more common in women, in the elderly, in people living in iodine-deficient areas, and in populations exposed to ionizing radiation [19].

In our cohort 51% (25/49) of the patients had thyroid nodules: 23 cases were revealed in the ultrasonography, and 2 patients underwent thyroidectomy due to nodular goiter. In 10 patients, only minor cysts with no clinical significance (less than 5 mm) were found and 13 patients had benign solid or cystic-solid lesions. According to American Thyroid Association Management Guidelines for Adult Patients with Thyroid Nodules, nodules <1 cm with very low suspicion ultrasonographic pattern and pure cysts do not require routine sonographic follow-up [13]. Therefore, pure cysts <1 cm are often not even described by specialists performing ultrasound examinations. As we studied ADPKD patients, we paid more attention to thyroid cysts and reported all of them, although they were all below 5 mm and were not clinically relevant. For this reason, we believe that these small purely cystic lesions in the thyroid gland can be ignored when comparing thyroid nodules to the population studies. Not taking into account those minor thyroid cystic lesions, the prevalence of thyroid gland nodules in our cohort (22%) is close to data reported in north Poland (23.7%) [14], southern Finland (27.0%) [20], and Belgium (19.0%) [21], where iodine deficiency is low.

Unexpectedly, we observed a high percentage of ADPKD patients with elevated levels of anti-thyroid antibodies. 42.86% of our patients had elevated TgAb, and 16.33% had positive TPOAb. Depending on the studied population, both, TPOAb and TgAb, are detected in 10–15% of patients with non-thyroid immune disorders [22]. So far, no elevated levels of anti-thyroid antibodies in ADPKD patients have been reported and this phenomenon has not been investigated.

Interestingly, loss-of-function of primary cilia on an animal model has been recently found to contribute to a malignant transformation from degenerated thyroid follicles. Mice devoid of primary cilia showed normal folliculogenesis and hormonogenesis, however, with increasing age, follicular cells formed papillary and solid proliferative nodules with characteristic nuclear features of human thyroid carcinomas [23]. Furthermore, according to a leading evidence-based clinical decision support resource, cysts in ADPKD patients may grow not only in kidneys but also in other organs, including the liver, pancreas, thyroid gland, and/or spleen [12].

However, based on our study and the PubMed literature review, we found no convincing proof for the hypothesis that ADPKD patients have an increased incidence of thyroid lesions. Ha et al. reported thyroid cysts in only two (6.7%) of their series of thirty patients with ADPKD, attributing this finding to extra-kidney manifestation [10]. Alejmi et al. described a 44-year-old female ADPKD patient with end-stage renal failure and multiple, large (above 1 cm) thyroid cysts [11]. In our cohort, no patient occurred to have multiple cystic nodules affecting both thyroid lobes as in the described patient. Moreover, Cuna et al., in their retrospective study on 2,147 patients with end-stage renal disease, 20.1% of whom had ADPKD, observed that 41.5% of their ADPKD patients had a goiter. Besides, ADPKD was found to be associated with hypothyroidism. The study did not include thyroid cysts [8]. It should be highlighted that the higher prevalence of hypothyroidism and nodular thyroid disease may result from end-stage renal disease and hemodialysis rather than ADPKD. Several studies indicated that benign and malignant thyroid nodules are more common in patients with end-stage renal disease than in the general population [6, 7]. Therefore, to avoid the influence of renal failure on the number of thyroid nodules, we did not enroll ADPKD patients with abnormal renal function to our study.

ADPKD is a systemic ciliopathy that presents with renal and extrarenal manifestation following reduction in levels of PC1 and PC2 below a critical threshold as a result of mutation in the genes *PKD1* and *PKD2* [24]. If the mechanism of cilia involvement in the pathogenesis of thyroid nodules is correct, fast progressors with kidney length above 16,5cm early in ADPKD [4], should have theoretically higher probability to have low PC1/PC2 and more thyroid nodules. However, we did not observe statistically significant differences in the number of thyroid nodules depending on the rate of renal progression. Plausibly, mechanisms other than defective cilia signaling are involved in the risk for focal thyroid lesions formation in human [9].

There were several limitations in our study. First of all, its small size and use of a sample from a single ethnic group. Secondly, no control group. Although we compared the results to the population study from the region, different ultrasound systems and another consultant performing the examination may have led to potential bias. The ultrasonography is a subjective examination, and distinct ultrasound machines can lead to slightly different measurements. Presumably, the smaller volumes of our ADPKD patients' thyroids and thyroid nodules in comparison to the population study are due to these limitations. Moreover, the serum TSH concentration in Karaszewski et al. study was determined using the enzyme immunoassay (EIA) method (Roche Laboratories Poland) [14]. Hence, the lower level of serum TSH in our ADPKD patients compared to the population study might be due to the different analytical methods. Furthermore, Karaszewski et al. examined individuals with no thyroid diseases and omitted the description of small cysts that are of no clinical significance [14]. Therefore, to avoid potential bias, further reduction of the study group was necessary. We excluded from our cohort, for the comparison with the population study, all patients with past medical history positive for thyroid diseases, and we did not take into account in our ADPKD patients minor cystic lesions without clinical significance. None of our patients had a malignant change in the thyroid gland. However, as thyroid cancers are exceedingly rare, our cohort was not large enough to comment on the prevalence of thyroid cancer in ADPKD patients.

In conclusion, despite its pathogenic plausibility, we have not confirmed higher incidence of solid or cystic-solid thyroid gland lesions, including neoplasia, in early ADPKD patients. Further research is required to determine whether there is a greater incidence of cystic lesions in ADPKD patients. Moreover, the rate of progression of kidney function decline seems to be not accompanied by a higher incidence of thyroid pathology.

## Data Availability

All data generated or analysed during this study are included in this published article.
